# Public Attitudes Toward Guided Internet-Based Therapies: Web-Based Survey Study

**DOI:** 10.2196/10735

**Published:** 2018-05-15

**Authors:** Jennifer Apolinário-Hagen, Mathias Harrer, Fanny Kählke, Lara Fritsche, Christel Salewski, David Daniel Ebert

**Affiliations:** ^1^ Institute of Psychology Department of Health Psychology University of Hagen Hagen Germany; ^2^ Department of Clinical Psychology and Psychotherapy Alexander-University Erlangen-Nuremberg Erlangen Germany

**Keywords:** mental health, eHealth, attitude to computers, patient preference, cognitive therapy, acceptability of health care, stress, psychological, object attachment

## Abstract

**Background:**

Internet interventions have been proposed to improve the accessibility and use of evidence-based psychological treatments. However, little is known about attitudes toward such treatments, which can be an important barrier to their use.

**Objective:**

This study aimed to (1) determine attitudes toward guided internet interventions, (2) assess its acceptability compared with other internet-based formats, and (3) explore predictors of acceptance.

**Methods:**

A convenience-sample Web-based survey (N=646) assessed attitudes toward guided internet therapies (ie, perceived usefulness and helpfulness, and advantage relative to face-to-face therapy), preferences for delivery modes (ie, e-preference: guided internet interventions, unguided internet interventions, or videoconferencing psychotherapy), and potential predictors of attitudes and preferences: sociodemographics, help-seeking–related variables, attachment style, and perceived stress.

**Results:**

Although most participants perceived internet interventions as useful or helpful (426/646, 65.9%), a few indicated their advantage relative to face-to-face therapy (56/646, 8.7%). Most participants preferred guided internet interventions (252/646, 39.0%) over videoconferencing psychotherapy (147/646, 22.8%), unguided internet interventions (124/646, 19.2%), and not using internet interventions (121/646, 18.8%; missing data: 1/646, 0.2%). Attachment avoidance and stress were related to e-preference (all *P*<.05). Moreover, preference for therapist-guided internet interventions was higher for individuals who were aware of internet-based treatment (χ^2^_6_=12.8; *P*=.046).

**Conclusions:**

Participants assessed therapist-guided internet interventions as helpful, but not equivalent to face-to-face therapies. The vast majority (523/646, 81.0%) of the participants were potentially willing to use internet-based approaches. In lieu of providing patients with only one specific low-intensity treatment, implementation concepts should offer several options, including guided internet interventions, but not limited to them. Conversely, our results also indicate that efforts should focus on increasing public knowledge about internet interventions, including information about their effectiveness, to promote acceptance and uptake.

## Introduction

### Background

With 12-month prevalences ranging across countries from 9.8% to 19.1% [1], mental health disorders are widespread. Mental health disorders constitute one of the leading causes of disability [2] and are associated with low quality of life, increased risk of developing chronic physical conditions and related mortality [3,4], and an immense economic burden leading to productivity losses and substantial societal costs [5,6]. Yet fewer than half of individuals affected by mental health disorders are detected and receive professional treatment [7]. Untreated mental illness is estimated to account for 13% of the total global burden of disease [8]. Structural barriers such as limited access to treatment have been named as a reason for the insufficient uptake of individuals with mental health disorders [7]. Additionally, attitudinal barriers, such as personal stigma [9] or preferring to solve problems on one’s own, may be decisive in explaining insufficient treatment rates [10].

Using the internet as a delivery mode for self-help treatments has thus been discussed as a promising chance to inform the dissemination of professional treatment, as electronic mental health services (eMHSs) allow for mass deliverance of anonymous, low-threshold treatment options that may reach individuals for whom traditional face-to-face approaches are not an option [11,12]. In recent years, a large number of randomized controlled trials have shown that internet interventions can effectively treat various mental health disorders, such as depression [13,14], anxiety [15-17], insomnia [18], alcohol use disorder [19], comorbid mental health problems in chronic somatic diseases [20,21], and psychosomatic disorders [22]. The largest evidence base exists for the effectiveness of guided interventions [22,23], and research has shown that such approaches can be effective when delivered under routine care conditions [24-26]. In addition to guided or unguided internet interventions, videoconferencing psychotherapy (VCP) is considered as a further option to overcome regional barriers for a variety of patient populations [27]. However, the poor adoption of eMHSs worldwide indicates that low acceptability and intention to use might constitute a barrier in reaching the full potential of internet-based approaches (cf, [28-32]).

### Public Acceptance Indicators for E-Mental Health Services

Determinants of intentions to use eMHSs are not well understood [28,29]. Yet there are indicators commonly discussed as influential for help-seeking intentions and acceptance of eMHSs, such as attitudes [33-35] and “e-preferences” [28].

#### Attitudes

Positive public attitudes could be an indicator of acceptance and adoption of internet interventions. Generally, attitudes can be characterized as an aggregate of subjective assessments about an object, ranging, for example, from harmful to helpful [36].

The theory of planned behavior [37] proposes that attitudes, among other factors, shape individuals’ intentions, which then lead to a certain behavior. Individuals’ personal expectancies are assumed to shape such attitudes and to thereby influence behavioral intentions [37]. In accordance with the unified theory of acceptance and use of technology [38], performance expectancy (ie, how useful an individual perceives an intervention to be for reaching a specific goal) might thus play an important role in the adoption and acceptance of internet interventions [39,40] and provide a guideline in overcoming the limitations in the acceptability of eMHSs [34,39,41,42].

#### E-Preference

Technology acceptance of eMHS can be operationalized by intentions to use these services [39], which can be affected by the individual preference for a specific delivery mode [28]. Treatment preference means to choose a treatment in favor of an alternative option. Research evidence suggests that considering patients’ preferences for a psychological treatment is associated with improved clinical outcomes [43]. However, little is known about preferences for specific delivery modes, such as therapist-guided treatment, unguided internet interventions, and VCP, and their impact on the willingness to use eMHSs. Some studies identified a preference for traditional (face-to-face) over internet-based treatment (eg, [28,29,31,44-47]) and for therapist-guided over unguided eMHSs (eg, [28,29,33]).

#### Determinants of Attitudes Toward and Preferences for Internet-Based Therapies

Potential determinants of attitudes and preferences for eMHS include sociodemographics such as age, region [45], or professional background [48-50]. Regarding health-related and help-seeking variables, using the internet for mental health information [45,51], previous use of eMHSs [31], a history of mental illness and help-seeking experience such as undergoing psychotherapy [29], knowledge about eMHSs or awareness of electronic therapies (“e-awareness”) [33,41,42], personality traits [28], and perceived stress [42,52] have been reported as predictors. Regarding the role of symptom severity, a recent study [12] illustrated a help-seeking behavior paradox in students, where individuals’ readiness to seek help from face-to-face services declined with increased perceived stress. In contrast, the same study also demonstrated a positive association between distress and seeking help online.

Attachment style may be a further predictor of eMHS uptake [52], since attachment theory [53] has been applied to predict intentions to use face-to-face help services (eg, [54]). Based on early infant-caregiver interactions, relatively stable internal working models of the self and others in terms of mental representations of close relationships are built. These implicit expectations regarding self-efficacy and reliance on significant others in stressful situations are manifested in adulthood [53]. Adult attachment style can play a role in preferences and attitudes toward seeking help in the context of emotionally relevant relationships, such as in mental health care [52,54]. While a secure attachment style (low attachment anxiety and avoidance; ie, positive models of the self and others) is associated with functional coping strategies, insecure attachment styles were identified as a global vulnerability factor for mental health [55,56] and are related to altered stress responses, symptom reporting, and less use of health care resources [57,58]. However, the role of attachment styles in the readiness to use eMHSs remains unclear [52], especially concerning different delivery modes of internet interventions that vary in the degree of human support.

Taken together, the identification of determinants of attitudes toward and preferences for eMHSs is at an early stage [34]. This study addressed this research gap.

### Objective

The purpose of this study was to (1) explore attitudes toward guided internet interventions and to (2) assess the acceptability of guided internet interventions compared with other formats of internet-based delivery (ie, e-preference: unguided self-help interventions and VCP vs not using eMHSs in case of emotional problems). Another goal was to (3) identify determinants of the public acceptability of eMHSs by exploring associations between attitudes toward guided internet interventions, preferences for a specific delivery mode of eMHSs, and participant characteristics (ie, sociodemographics, help-seeking–related variables, attachment style, and perceived stress).

## Methods

### Study Design and Participants

We conducted a cross-sectional Web-based survey using a quasi-experimental study design. Data were collected between November 2015 and June 2016 using Unipark software (Enterprise Feedback Suite survey, version 10.6, Questback). We obtained a convenience sample (N=646) via the virtual laboratory and Moodle of the University of Hagen, Hagen, Germany, and social media websites (Facebook, Facebook Inc; and Xing, Xing AG). No ethical approval was required. Inclusion criteria were self-reported age over 18 years and written informed consent. Psychology students could receive credits for their participation.

### Measures

#### Attitudes Toward Guided Internet Interventions

We used a modified 17-item version of an e-therapy attitudes measure (ETAM) [42] containing statements about typically cited benefits of internet therapy and its comparability with face-to-face psychotherapy, as well as subjective beliefs (eg, about data security). Participants were asked to rate their agreement with each statement on a 5-point rating scale ranging from 0 (“strongly disagree”) to 4 (“strongly agree”). To ensure comparability, participants were instructed to rate items regarding guided internet interventions (see Textbox 1). Based on previous exploratory factor analysis, we identified two factors, which we termed perceived usefulness and helpfulness, and advantage relative to face-to-face therapy. Multimedia Appendix 1 provides detailed information about the exploratory factor analysis. For classification of attitudes, we used predefined cutoffs in line with previous work using the ETAM [41,42]: mean scores <1.5 (a median score of 0 or 1) were defined as negative, values between 1.5 and 2.49 (median score of 2) as neutral, and scores ≥2.5 (median scores of 3 or 4) as positive attitudes toward guided internet interventions. Cronbach alpha was excellent in this survey (alpha=.92).

#### Preference for Internet Interventions (E-Preference)

We operationalized preference (see Textbox 1) by assessing help-seeking intentions for different delivery modes of internet interventions (e-preference, options 1-3) in contrast to the disinclination to use internet interventions in case of emotional problems (non–e-preference, option 4).

#### Determinants of Attitudes Toward and Preferences for Internet Interventions

##### Sociodemographics and Help-Seeking–Related Variables

Sociodemographic characteristics were sex, age, marital status, native language, region, country of residence, educational level, employment status, and work in health care or the social sector.

**Preference for internet therapies illustrated by case vignettes. Options 1-3: internet therapies differentiated by the degree of professional support. The instruction was adapted and translated from German.**

Internet-based psychotherapies include a broad spectrum of types of treatments for relatively well treatable, mild to moderate forms of depression and anxiety disorders. The following 3 examples illustrate distinct types of internet therapies:

1. Unguided internet-based self-help treatment programs: The patient follows a Web-based, structured self-help treatment program, including problem-specific tasks, exercises, and tutorials, via mobile phone or computer for several weeks without individual feedback.
2. Therapist-guided internet-based self-help treatment programs: The patient follows a Web-based, structured self-help treatment program with therapist guidance. Communication with the therapist consists of text-based feedback via email or chat provided on demand.
3. Videoconferencing psychotherapy: The communication is mediated by a webcam. As with face-to-face psychotherapy, the treatment takes place within specified sessions with immediate verbal and nonverbal feedback.

In case of emotional problems, which of the described interventions would you most likely personally use? Please choose the internet therapy form you prefer most based on your current expectations.

1. Unguided internet-based self-help treatment
2. Therapist-guided internet-based self-help treatment
3. Videoconferencing psychotherapy
4. I would not use any internet therapy at all

We investigated participants’ awareness of electronic therapy (e-awareness) by asking them whether they had ever heard or read about internet-based therapies. We also asked participants to assess their subjective health status, experiences with online counseling or conventional inpatient or outpatient psychotherapy, and their frequency of seeking health information online.

##### Attachment Style

We considered attachment style as a potential determinant of the acceptance of guided internet interventions, since previous work indicated a connection between individual needs for interpersonal proximity versus distance in case of emotional problems and help-seeking intentions (cf, [54]). We measured adult attachment using the Experiences in Close Relationships-Relationship Structures questionnaire [59] 9-item global version to assess attachment anxiety and avoidance [60]. Participants were asked to rate the extent to which they believed each statement best described their feelings about close relationships on a 7-point Likert scale ranging from 1 (“strongly disagree”) to 7 (“strongly agree”). The intercorrelation of dimensions (ρ_(646)_=.272, *P*<.001) was comparable with other studies [61]. Cronbach alpha was good for attachment avoidance (alpha=.88) and excellent for attachment anxiety (alpha=.91).

#### Assessment of Stress Perceptions

##### Current Stress Level

We used a visual analog scale [62] to assess current perceived stress level on a scale with 2 end points: 0 (“not at all”) and 10 (“maximum”).

##### Perceived Stress (Past Month)

To measure stress perceptions during the past 4 weeks, the we used the Perceived Stress Questionnaire 20-item short version (PSQ-20) [63]. Participants were asked to indicate how often statements applied to themselves on a 4-point Likert scale ranging from 1 (“almost never”) to 4 (“usually”). Cronbach alpha was poor (alpha=.55).

### Procedure

After entering the Web-based survey, participants were provided with the study information and consent form. Next, they were asked sociodemographic and help-seeking questions. Then, preference for specific forms of internet interventions, attitudes toward guided internet interventions, stress perceptions, and attachment style were assessed. The average completion time ranged from 10 to 15 minutes.

### Statistical Analysis

We considered only completed surveys for data analyses. To ensure data quality, data validation checks were performed independently by 2 researchers prior to the statistical analyses. Descriptive analyses were used to classify attitudes toward and preference for a specific delivery mode of internet therapies. Regarding predictors of attitudes, we explored differences in variance (analysis of variance) in attitudes (overall mean score) based on sociodemographics, health variables, and e-preference. Due to the scarce theory base and questionable multivariate normal distribution, we used Spearman rank correlation (ρ coefficient) instead of multiple regression analysis to identify associations between attitudes, attachment style, and stress perceptions. Moreover, we explored differences using 1-way analysis of variance and Pearson chi-square tests in preferences based on the same predictors as for attitudes. Pairwise comparisons (post hoc tests) to examine mean differences (M_diff_) were conducted using Bonferroni adjustments in case of variance homogeneity (Levene test, *P*>.05) or Dunnett *C* test in case of variance heterogeneity. Statistical tests for significance (2-tailed hypotheses with alpha level of .05) were performed using IBM SPSS version 24 (IBM Analytics).

## Results

### Descriptive Analyses

Of 1300 respondents who accessed the platform, 778 provided informed consent, with 1 person declining and thus being excluded. We consequently analyzed the responses of 646 respondents who completed the survey. Tables 1 and 2 summarize the sample’s characteristics.

### Attitudes Toward Guided Internet Interventions

Analysis of attitudes toward guided internet interventions indicated an overall moderate acceptance (ETAM overall mean score, Table 3). As Table 4 shows, descriptive analyses further showed that, although most participants (426/646, 65.9%) perceived internet approaches as useful or helpful, only a few participants (56/646, 8.7%) also indicated that guided internet-based approaches had a relative advantage over or comparability with conventional face-to-face approaches.

Overall, participants agreed with 7 of the 17 positive statements about internet interventions made in ETAM items (Table 3). Those positively attributed beliefs about internet therapies involved modernity (item 1), compatibility with everyday life (item 3), accessibility (item 5), coverage of costs by health insurance providers (item 6), helpfulness (item 12), anonymity (item 14), and a chance to get help earlier (item 15).

Furthermore, 7 of the 17 items were classified as negative. Participants rather disagreed with the possibility of replacing face-to-face therapies (item 2), the equivalence of delivery modes (item 4), comparability of effectiveness (item 7) and therapeutic relationships (item 8), preference for internet therapy over face-to-face therapy (item 11), data security (item 13) and suitability for diverse populations (item 17).

Participants classified 3 items as neutral or undecided. These statements addressed internet therapies as an alternative to face-to-face therapies (item 9), willingness to use internet therapies (item 10), and the occurrence of misunderstandings (item 16).

### Preference for Different Delivery Modes

**Figure 1:**
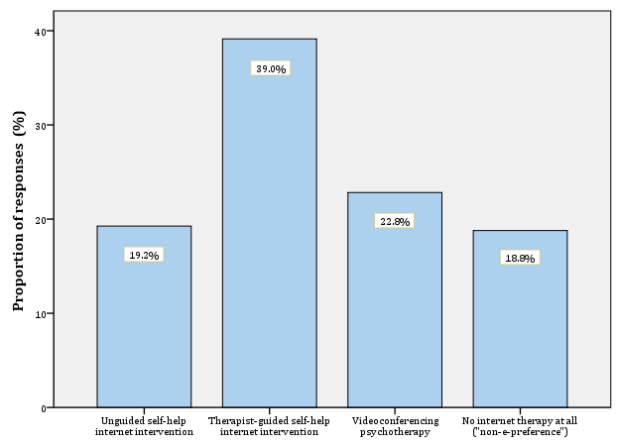
Participants' stated preference for a specific delivery mode of internet interventions (N=646).

As Figure 1 shows, most respondents indicated that they preferred guided internet interventions (252/646, 39.0%) over VCP (147/646, 22.8%), unguided internet interventions (124/646, 19.2%), or no Web-based treatment (121/646, 18.8%; missing data: 1/646, 0.2%). Thus, the vast majority were “e-preferers” (523/646, 81.0%).

**Table 1:** Sociodemographic characteristics (N=646).

Variables		Data
**Age (years)**		
	Mean (SD)	31.18 (10.08)
	Range (median)	18-64 (29)
**Sex, n (%)**		
	Female	493 (76.3)
	Male	147 (22.8)
	Other	3 (0.5)
	Missing data	3 (0.5)
**Native language, n (%)**		
	German	568 (87.9)
	Bilingual including German	40 (6.2)
	Other than German	37 (5.7)
	Missing data	1 (0.2)
**Marital status, n (%)**		
	Single	327 (50.6)
	Married or living in a close relationship	288 (44.6)
	Divorced or living separated	26 (4.0)
	Other	3 (0.5)
	Missing data	2 (0.3)
**Employment status^a^, n (%)**		
	Employed	177 (27.4)
	University student, part-time or full-time	345 (53.4)
	Employee in training^b^	14 (2.2)
	Self-employed	46 (7.1)
	Unemployed	11 (1.7)
	Parental leave	15 (2.3)
	Retired	8 (1.2)
	Current work incapability	3 (0.5)
	Other employment (commentary section)	27 (4.2)
**Employment in health care or social sector, n (%)**		
	No	493 (76.3)
	Yes, in a therapeutic field	57 (8.8)
	Yes, in a nontherapeutic field	96 (14.9)
**Education level attained, n (%)**		
	No school certificate	3 (0.5)
	Basic school qualification^c^	10 (1.5)
	Secondary school (*Mittlere Reife*)^d^	57 (8.8)
	German *Abitur* or *Fachabitur*^e^	329 (50.9)
	University degree (Bachelor level)	86 (13.3)
	University degree (Masters level)	143 (22.1)
	Postgraduate or postdoctoral degree	9 (1.4)
	Other degree (commentary section)	9 (1.4)
**Country of residence, n (%)**		
	Germany	585 (90.6)
	Other country^f^	60 (9.3)
	Missing data	1 (0.2)
**Region of residence, n (%)**		
	Village or small town, with <5000 inhabitants	100 (15.5)
	Provincial town, with 5000-19,999 inhabitants	99 (15.3)
	Medium-sized city, with 20,000-100,000 inhabitants	124 (19.2)
	Big city or metropolis, with >100,000 habitants	322 (49.8)
	Missing data	1 (0.2)

^a^The main employment was requested (if respondents had multiple roles, they were asked to choose in which role they spent most of their working time at the time of participation in this survey).

^b^German dual system: occupational trainee or pupil (secondary education).

^c^Basic school qualification with usually 9 school years of education in Germany (German *Hauptschule*).

^d^Secondary school (German *Mittlere Reife*), or 10 years of education in Germany.

^e^German *Abitur* or *Fachabitur* with 12-13 years of education in Germany. This education is necessary to get access to a college or university.

^f^Most of the subgroup of non-Germany residents indicated they lived in Austria (35/646, 5.5%).

### Determinants of Attitudes

#### Sociodemographic Variables

Attitudes and age were significantly and positively correlated (*ρ*_(643)_=.079, *P*=.045), with older participants displaying more favorable attitudes than younger participants toward internet-based guided self-help. Unemployed participants (mean 2.30, SD 0.69) showed more positive attitudes than employees in training (mean 1.37, SD 0.37, M_diff_ 0.93, SE 0.288, 95% CI 0.003-1.85). We found no significant differences in attitudes for sex, marital status, region, native language, education level, or work in the health care or social sector (all *P*>.05).

#### Help-Seeking–Related Variables

Frequency of seeking health information online was associated with differences in internet intervention attitudes (*F*_4,641_=6.675; *P*<.001, *η*_p_^2^=.040), with more positive attitudes reported by individuals who sought information weekly (mean 2.01, SD 0.61, M_diff_ –0.36, SE 0.112, 95% CI –0.67 to –0.04), several times a month (mean 2.11, SD 0.78, *M*_diff_ –0.45, SE 0.097, 95% CI –0.72 to –0.18), or rarely (mean 1.91, SD=0.59, M_diff_ –0.26, SE 0.080, 95% CI –0.48 to –0.04; all *P*<.05) than by those who never did (mean 1.66, SD 0.59). This was not the case for participants who reported seeking information daily (mean 1.46, SD 0.99); both groups (never, daily) expressed rather negative attitudes. There was a significant positive correlation between attitudes toward guided internet interventions and perceived stress on the PSQ-20 (*ρ*_(643)_=.092, *P*=.020). No significant differences in attitudes were identified for any of the other help-seeking–related variables (eg, e-awareness, attachment style, all *P*>.05).

### Determinants of E-Preference

#### Sociodemographic Variables

We found no significant differences in e-preferences (preference for guided or unguided internet interventions and VCP) based on age, sex, marital status, region, native language, education level, employment status, or work in the health care or social sector (all *P*>.05).

#### Help-Seeking–Related Variables

E-awareness significantly predicted a preference for different forms of internet-based therapy (χ^2^_6_=12.8; *P*=.046). Individuals who were aware of internet therapies (97/214, 45.5%) or not sure (40/87, 46.0%) were more likely to prefer guided internet interventions than were those who were not aware (115/343, 33.5%).

We found differences in e-preference based on experience with online counseling (χ^2^_3_=13.8; *P*=.003); persons with experience were less likely to prefer unguided interventions (7/68, 10.6%) than were those without (117/578, 20.2%).

Experience with psychotherapy also predicted e-preference (χ^2^_9_=21.6; *P*=.01). A preference for guided internet interventions was most common among persons without experience who were currently seeking a therapist (20/35, 57.1%) and persons with experience with psychotherapy (99/228, 43.4%). All subgroups were nonetheless most likely to prefer guided internet interventions.

**Table 2:** Sample characteristics (N=646).

Help-seeking–related variables		Data
**Subjective health status, n (%)**		
	Healthy or relatively healthy	492 (76.2)
	Acute illness	39 (6.0)
	Chronic illness	83 (12.8)
	Other (commentary section)	30 (4.6)
	Missing data	2 (0.3)
**Experience with online counseling, n (%)**		
	No, no experience with online counseling	578 (89.6)
	Yes, experience with online counseling	67 (10.4)
	Missing data	1 (0.2)
**Experience with psychotherapy^a^, n (%)**		
	No, I have no experience and I have also no need for psychotherapeutic help	303 (46.9)
	No, I have no experience, but I am seeking psychotherapeutic help from a therapist	35 (5.4)
	Yes, I am in therapy	79 (12.2)
	Yes, in the past (experience with psychotherapy)	228 (35.3)
	Missing data	1 (0.2)
**Web-based health information use (frequency), n (%)**		
	Daily	15 (2.3)
	Several times a week	49 (7.6)
	Several times a month	146 (22.6)
	Rarely or occasionally	369 (57.1)
	Very rare or never	67 (10.4)
**E-awareness (awareness of the existence of electronic therapies), n (%)**		
	No (not aware)	343 (53.1)
	Yes (aware)	214 (33.1)
	Not sure	87 (13.8)
	Missing data	2 (0.4)
**Attachment style (ECR-RS^b^), mean (SD), median, range**		
	Attachment avoidance	3.61 (1.38), 3.50 (1.00-7.17)
	Attachment anxiety	3.46 (1.77), 3.33 (0.00-7.00)
**Stress perceptions, mean (SD), median (range)**		
	Current stress (visual analog scale)	5.67 (2.96), 6.0 (0-10)
	Perceived stress (Perceived Stress Questionnaire 20-item short version)	47.00 (19.60), 46.67 (3.33-95.00)

^a^Experience with psychotherapy or current need or demand for professional psychological help.

^b^ECR-RS: Experiences in Close Relationships-Relationships Structures questionnaire.

**Table 3:** Summary of attitude assessment results with the e-therapy attitudes measure^a^ (ETAM; N=646).

ETAM			Mean (SD)	Median	Range
Overall score attitude assessment (ETAM mean score)			1.93 (0.72)	1.94	0-4
Perceived usefulness and helpfulness (PU) scale			2.72 (0.79)	2.86	0-4
Relative advantage and comparability (RA) scale			1.37 (0.78)	1.33	0-4
**Scale item^b^**					
	1./PU	Internet-based therapies are modern and in line with our modern times.	2.84 (1.04)	3.0	0-4
	2./RA	Internet-based therapies will replace conventional face-to-face psychotherapy in the future.	0.94 (0.93)	1.0	0-4
	3./PU	Internet-based therapy is more compatible with work and private life than conventional face-to-face therapy.	2.73 (1.05)	3.0	0-4
	4./RA	It makes no difference to me whether psychotherapy is conducted through the internet or in a psychotherapy practice in a clinic.	0.69 (0.96)	0.0	0-4
	5./PU	Internet-based therapies will reach more individuals with mental health problems.	2.77 (1.09)	3.0	0-4
	6./PU	Health insurance companies should cover the costs for internet-based therapies.	2.77 (1.16)	3.0	0-4
	7./RA	Internet-based therapy programs are as effective as conventional face-to-face psychotherapies.	1.39 (0.99)	1.0	0-4
	8./RA	Trust in a therapist can be just as easily built on the internet as in conventional face-to-face psychotherapy.	1.39 (1.14)	1.0	0-4
	9./RA	Internet-based therapies are an appropriate alternative to conventional face-to-face psychotherapy.	1.82 (1.10)	2.0	0-4
	10./RA	In case of mental health problems, I would attend an internet-based therapy.	1.70 (1.34)	2.0	0-4
	11./RA	I would prefer an internet-based therapy to a conventional face-to-face psychotherapy.	1.03 (1.16)	1.0	0-4
	12./PU	Internet-based therapies will reach more patients and help them.	2.55 (1.10)	3.0	0-4
	13./RA	I’m not particularly worried about data security in internet therapies.	1.29 (1.33)	1.0	0-4
	14./PU	The anonymity in internet therapies decreases the threshold to speak openly and honestly about important issues.	2.69 (1.19)	3.0	0-4
	15./PU	Through the dissemination of internet therapies, persons will get professional help earlier.	2.75 (1.04)	3.0	0-4
	16./RA	Misunderstandings occur in internet therapies as often as in conventional psychotherapies.	1.96 (1.23)	2.0	0-4
	17./RA	Internet therapies are suitable for most patients, regardless of their personal background (age, sex, education, etc).	1.54 (1.18)	1.0	0-4

^a^The ETAM rating scale ranged from 0 (“strongly disagree”) to 4 (“strongly agree”).

^b^All items were translated from German to English. Item 1 refers to expectations and can be interpreted best in connection to other attitudinal items (compared with the previous version with 14 items, items 1-12 remained and items 13-17 are novel items of the 17-item version).

**Table 4:** Classification of attitudes toward guided internet interventions assessed by the e-therapy attitudes measure (ETAM; N=646).

ETAM	Classification of ETAM scores^a^		
	Low acceptance, negative attitude, n (%)	Moderate acceptance, neutral attitude, n (%)	High acceptance, positive attitude, n (%)
Overall mean score (attitudes)	168 (26.0)	324 (50.2)	124 (23.8)
Perceived usefulness and helpfulness	44 (6.8)	175 (27.1)	426 (65.9)
Relative advantage and comparability	364 (56.3)	226 (35.0)	56 (8.7)

^a^Low acceptance, negative attitude: scale mean score range 0-1.49; moderate acceptance, neutral attitude: scale mean score range 1.5-2.49; high acceptance, positive attitude: scale mean score range 2.5-4.0.

##### Attachment Style

Attachment avoidance significantly predicted e-preference (*F*_3,640_=6.315; *P*<.001, *η*_p_^2^=.029). Participants with higher attachment avoidance were less likely to prefer VCP than other formats (unguided internet interventions: M_diff_ –0.579, SE 0.166, 95% CI –1.020 to –0.139; guided internet interventions: M_diff_ –0.426, SE 0.142, 95% CI –0.801 to –0.051; non–e-preference: M_diff_ –0.651, SE 0.166, 95% CI –1.095 to –0.208). There was no significant association between preference and attachment anxiety (*F*_3,640_=2.247; *P*=.08).

##### Perceived Stress

Current perceived stress was associated with e-preference (visual analog scale: *F*_3,640_=3.855; *P*=.009, *η*_p_^2^=.018), with participants who experienced higher stress being more likely to prefer guided interventions than VCP (M_diff_ 0.86, SE 0.277, 95% CI 0.13-1.60). Scores for current stress were lowest in non–e-preferers (mean 5.26, SD 2.80) and highest in those who preferred guided internet intervention (mean 5.90, SD 2.60). Another significant difference between the preference groups was shown for perceived stress in the past month (PSQ-20: *F*_3,638_=2.943; *P*=.03, *η*_p_^2^=.014). Pairwise comparisons were not significant. PSQ-20 scores were lowest for non–e-preference (mean 44.39, SD 20.81) and highest for therapist-guided intervention preference (mean 49.67, SD=19.14).

The other help-seeking–related variables were not associated with significant differences in preferences (all *P*>.05).

## Discussion

### Principal Findings

#### Attitudes Toward Guided Internet Interventions

This study identified an overall moderate public acceptance, or moderately positive attitudes, toward guided internet interventions in a German sample. This tendency is in line with another study on a psychoeducational intervention using an adapted ETAM version [41]. Participants supported health care insurance coverage of costs for guided internet-based therapies and endorsed the helpfulness of such approaches, their perceived anonymity, and the chance to receive help earlier compared with traditional health care. At the same time, participants disagreed with the supposed comparability of guided internet interventions with face-to-face psychotherapy, for example, with regard to their effectiveness and possibility to develop a good therapeutic relationship. Our findings are, furthermore, consistent with earlier research with respect to a general preference for face-to-face therapies over internet interventions [28,29,44], data security concerns [64,65], and perceived higher compatibility of internet interventions with everyday life [11,29].

We found no relevant differences for sociodemographics as predictors of attitudes toward internet-based guided self-help. Interestingly, neither education level nor sex was associated with attitudes. These results are in line with a study on the acceptance of internet-based interventions in chronic pain [66]. Replication of this finding might indicate that the often-reported overrepresentation of woman and highly educated participants in randomized controlled trials evaluating internet interventions [13,67-72] might not be due to lower acceptance of digital health interventions in general, but due to other relevant barriers such as lower willingness to seek help. Future research should try to shed light on low utilization rates among persons with low education and men.

Moreover, participants with higher levels of perceived stress in the past month tended to express a more positive attitude toward internet interventions, which is consistent with a prior study using the same instrument to assess attitudes [42]. This might point to improved acceptance of such guided internet interventions among participants in a stressful situation with an actual need for support.

#### Preference for Specific Delivery Formats

This study identified a clear preference for guided over unguided internet interventions, which only few studies have investigated before [34]. Interestingly, guided internet interventions were also preferred over VCP. Approximately four-fifths of the participants were willing to use internet-based approaches for emotional problems, indicating a broad applicability of internet interventions for mental health care.

High e-awareness was associated with a preference for guided internet interventions. Overall, e-awareness in our sample was low (33.1%), which could be, for instance, seen in context of the early stage of implementation of eMHSs in Germany [50,73] and might rise further in the future. This is supported by previous German surveys reporting even lower rates of e-awareness (14.0%-27.3%; [42,52,64]), including a representative socioeconomic panel (SOEP-Innovation Modules 2016, N=4802) showing 24.4% e-awareness (D Richter, written communication, May 2017). Experience with seeking psychological help formats was also a determinant of preferences, which is consistent with other studies [29,31]. Results also suggest that attachment avoidance was associated with a higher preference for guided and unguided self-help via internet interventions, and very low preference for VCP. This finding contributes to research on links between attachment styles and face-to-face health care use readiness [52,57,58] and might indicate that internet-based (guided) self-help approaches could help to reach individuals for whom attitudinal and other psychological barriers such as attachment avoidance might be a drawback for use of an intervention [74].

Furthermore, participants with higher levels of perceived stress showed a higher preference for internet-based guided self-help than for VCP. This might indicate that individuals with stressful lives have problems adhering to fixed synchronous therapy sessions, and that providing asynchronous treatments might help them to get access to psychological treatments, which they would otherwise not use. Such an assumption is supported by studies that found high proportions of first-time help seekers in internet-based stress management programs [75-79]. However, future research is needed to confirm such an assumption.

### Implications

This study provides several important implications for research and practice.

#### Providing Asynchronous Treatment Formats to Increase Health Care Utilization

First, results indicate that, although internet-based approaches are not an option for some individuals, a large proportion of participants in this study were potentially inclined to use eMHSs for treatment. However, e-preference rates were lowest for VCP, which, as a synchronous delivery format, is the most similar to conventional face-to-face psychotherapy [27]. Results also indicated that individuals with high attachment avoidance were least inclined to use this synchronous format to seek help, but were more willing to use asynchronous internet-based interventions. Perceived stigma and a preference for managing mental health problems on one’s own are known barriers to seeking synchronous treatment [10], and personal characteristics such as attachment style may contribute [57,58]. This suggests that provision of asynchronous treatment options, such as guided or unguided internet interventions, could be a feasible way to reach larger proportions of the general population, especially individuals who would not use synchronous options such as face-to-face psychotherapy or VCP. Matched-care models have been proposed before (cf, [80]), allocating internet-based or face-to-face treatment based on symptom severity; findings in this study, however, pointed out that various asynchronous as well as synchronous treatment formats should be provided simultaneously to reach as many individuals affected by mental health problems as possible.

Second, these results also suggested that offering guidance alongside internet-based self-help internet interventions in routine care could, from a public health perspective, have a major influence on their effects on a population level. Whether to offer guided or unguided interventions in routine care has been debated in the literature since internet-based self-help has emerged. This discussion has since predominately focused on potential differences in adherence, effects, and costs [22,81-83]. Meta-analytic findings clearly indicate that stand-alone guided self-help interventions can be effective in the prevention and treatment of a range of mental health problems, including depression [68], anxiety [72], and stress [77]. However, although more patients could potentially be treated for the same costs using unguided self-help, a basic prerequisite to exploiting the potential of any effective treatment is that affected individuals are willing to use it [84]. This study showed that approximately twice as many participants preferred guided interventions over unguided interventions. Thus, with evidence showing guided interventions to be comparable with face-to-face psychotherapy, for example for depression and anxiety [17,85], and with large effect sizes of guided formats when delivered under routine care conditions [25,86-88], preference should be given at the moment, whenever possible, to guided self-help in routine care. However, it should also be acknowledged that almost 20% of participants preferred unguided self-help; hence, future studies should clarify whether offering both guided and unguided interventions could lead to greater effects on a population level due to higher overall utilization rates, compared with offering only one of the two options.

#### Raising E-Awareness and Knowledge

Awareness about internet-based treatment was rather low in this sample, but was positively associated with higher preference for guided internet interventions. Furthermore, participants did not find internet interventions to be equal in effectiveness and therapeutic relationship to face-to-face therapies. Previous research, however, has shown that the effects of guided internet interventions are comparable with face-to-face therapies [17,85] and that therapeutic relationships are of the same quality as in conventional treatment [1-4]. This finding points to the importance of developing measures to increase awareness of and knowledge about the efficacy of internet-based treatment in the public to raise its acceptance. Acceptance-facilitating interventions using brief, highly scalable educational videos have been shown to be a valid strategy to enhance the acceptability of internet interventions in clinical practice [66,71,84]. As acceptance-facilitating interventions may be easily disseminated through official health care information channels, they might be an auspicious approach to increase e-awareness and knowledge concerning internet interventions, and thus raise their public acceptance.

### Limitations

First, the early stage of validation of the ETAM and the application of a heuristic rule to classify attitudes are a limitation that might have biased results regarding the categorization of attitudes with mainly neutral or undecided views. Future efforts should try to develop data-based cutoff values using representative samples. Second, the prior presentation of the case vignette regarding e-preferences might have led to more positive attitudes toward internet interventions, considering that a previous study using the ETAM without this case vignette revealed overall negative views [42]. Hence, these results might only be generalizable to situations in which potential participants receive minimal information about internet interventions. Third, we investigated determinants of attitudes toward and preferences for internet therapies based on self-reports, with most respondents (492/646, 75.2%) rating themselves as relatively healthy. It may be the case that attitudes toward digital mental health approaches change with current symptomatology, help-seeking wishes, and the availability of other formats for preferable treatments in routine care. Since the study was conducted in Germany, results may only be applicable to countries with similar economies or health care systems. Fourth, e-preferences were operationalized only regarding preferences for a specific treatment, and we do not know whether patients were nevertheless willing to use an alternative treatment format, if their preference would not be available in routine care, which should be tested in future studies. Moreover, with regard to non–e-preferers, we only assessed whether somebody would not prefer guided and unguided self-help or VCP, but we did not assess preference for face-to-face psychotherapy and pharmacotherapy, which should also be tested in subsequent studies.

### Conclusions

This study revealed moderately positive attitudes toward guided internet interventions and a clear public preference for guided over unguided internet-based treatment and VCP. Results of this survey indicated that increasing awareness about the existence of effective internet-based treatment options should be a key priority to raise their acceptability, and that guided internet-based programs should be implemented in routine care along with conventional face-to-face treatment to account for different patient preferences and help-seeking characteristics.

## Appendix

Multimedia Appendix 1 Exploratory factor analysis for the e-therapy attitudes measure (including pattern and structure matrices).

## Abbreviations

ECR-RS: Experiences in Close Relationships-Relationships Structures questionnaire
eMHS: electronic mental health service
ETAM: e-therapy attitudes measure
M_diff_: mean difference
PSQ-20: Perceived Stress Questionnaire 20-item short version
PU: perceived usefulness and helpfulness
RA: relative advantage and comparability
SOEP: socioeconomic panel
VCP: videoconferencing psychotherapy
