# Supplementary material for: Public Attitudes Toward Guided Internet-Based Therapies: Web-Based Survey Study
Source: JMIR Ment Health. 2018 May 15;5(2):e10735. doi: 10.2196/10735 (PMC5974457; doi:10.2196/10735)
Supplement: Multimedia Appendix 1 [file mental_v5i2e10735_app1.pdf]

Multimedia Appendix 1

Factor Analyses

For e-therapy attitudes measure (ETAM), we conducted an exploratory factor analysis (EFA) using maximum likelihood (MI) as extraction method and promax (with Kaiser Normalization) as oblique rotation procedure (κ=4). The Kaiser-Guttman criterion was applied to obtain the number of extracted factors. The suitability of data for conducting the EFA with oblique rotation was confirmed given both the results of the Bartlett sphericity test (χ^2_{136}=3320.468, *P*<.001) and the Kaiser-Meyer-Olkin (KMO) index (.935) for sampling adequacy. The EFA with the modified 17-item version of the measure resulted in a 2-factor solution (see Table 3 and 4). The total explained variance for the first two factors amounted to 53.43% (unrotated sum of square factor solutions with respect to the extraction/initial eigenvalues; "factor 1" with 44.25%, and "factor 2" with 9.18% of explained variance). The rotated sums of square factor solutions were 41.36% for "factor 1," and 6.16% for "factor 2" of explained variance. The rotated sums of squared loadings were as follows: 6.39 (factor 1), and 5.80 (factor 2). The factor correlation matrix revealed an intercorrelation of *r* = .693.

Table 4. Pattern matrix of the exploratory factor analysis (EFA) for the e-therapy attitude measure.

Items of the e-therapy attitude measure	Factors [a, b, c]	
	1. Relative Advantage (Comparability)	2. Perceived Usefulness (Helpfulness)
8. Trust in a therapist can be just as easily built on the Internet as in conventional face-to-face psychotherapy.	**.835**	–.102
7. Internet-based therapy programs are as effective as conventional face-to-face psychotherapy.	**.798**	+++
4. It makes no difference to me whether psychotherapy is conducted through the Internet or in a practice in a clinic.	**.748**	-.104
11. I would prefer an Internet-based therapy to a conventional psychotherapy.	**.719**	+++
9. Internet-based therapies are an appropriate alternative to conventional face-to-face psychotherapy.	**.715**	.145
2. Internet-based therapies will replace conventional face-to-face psychotherapy in the future.	**.678**	+++
10. In case of mental health problems, I would attend an Internet-based therapy.	**.576**	.291
17. Internet therapies are suitable for most patients, regardless of their personal background (age, gender, education, etc.)	**.509**	.101
16. Misunderstandings occur in Internet therapies as often as in conventional psychotherapies.	**.419**	+++
13. I´m not particularly worried about data security in Internet therapies.	**.363**	.160
5. Internet-based therapies will reach more people with mental health problems.	-.175	**.914**
12. Internet-based therapies will reach more patients and help them.	.108	**.748**
15. Through the dissemination of internet therapies, persons will earlier get professional help	+++	**.695**
3. Internet-based therapy is better compatible with work and private life than conventional face-to-face therapy.	+++	**.606**
14. The anonymity in Internet therapies decreases the threshold to speak openly and honestly about important issues.	+++	**.540**

6. Health insurance companies should cover the costs for Internet-based therapies.	.215	**.531**
1. Internet-based therapies are modern and in line with our modern times.	.126	**.522**

[a] Factor loadings smaller than .1 were suppressed (+++).
[b] Extraction method: principal axis factor analysis (maximum likelihood); rotation method: promax with Kaiser normalization. Item rotation converged in 6 iterations.
[c] Mapping of items to factor: bold values indicate that the highest factor loading on a factor.

Table 5. Structure matrix of the exploratory factor analysis (EFA) for the e-therapy attitude measure.

Items of the e-therapy attitude measure	Factors [a, b, c]	
	1. Relative advantage	2. Usefulness or helpfulness
9. Internet-based therapies are an appropriate alternative to conventional face-to-face psychotherapy.	**.816**	.641
7. Internet-based therapy programs are as effective as conventional face-to-face psychotherapy.	**.780**	.527
10. In case of mental health problems, I would attend an Internet-based therapy.	**.778**	.691
8. Trust in a therapist can be just as easily built on the Internet as in conventional face-to-face psychotherapy.	**.764**	.476
11. I would prefer an Internet-based therapy to a conventional psychotherapy.	**.743**	.534
4. It makes no difference to me whether psychotherapy is conducted through the Internet or in a practice in a clinic.	**.676**	.415
2. Internet-based therapies will replace conventional face-to-face psychotherapy in the future.	**.667**	.455
17. Internet therapies are suitable for most patients, regardless of their personal background (age, gender, education, etc.)	**.579**	.454
13. I´m not particularly worried about data security in Internet therapies.	**.474**	.412
16. Misunderstandings occur in Internet therapies as often as in conventional psychotherapies.	**.428**	.303
12. Internet-based therapies will reach more patients and help them.	.626	**.822**
5. Internet-based therapies will reach more people with mental health problems.	.458	**.792**
15. Through the dissemination of internet therapies, persons will earlier get professional help	.487	**.699**
6. Health insurance companies should cover the costs for Internet-based therapies.	.583	**.680**
3. Internet-based therapy is better compatible with work and private life than conventional face-to-face therapy.	.444	**.623**
1. Internet-based therapies are modern and in line with our modern times.	.489	**.610**
14. The anonymity in Internet therapies decreases the threshold to speak openly and honestly about important issues.	.337	**.514**

[a] Factor loadings smaller than .1 were suppressed (+++).
[b] Extraction method: principal axis factor analysis (maximum likelihood); rotation method: promax with Kaiser normalization. Item rotation converged in 6 iterations.
[c] Mapping of items to factor: bold values indicate that the highest factor loading on a factor.
